# Characterization of clear cell - Renal cell carcinoma using neutrophil - Lymphocyte ratio

**DOI:** 10.6026/973206300210499

**Published:** 2025-03-31

**Authors:** Santhosh S., Poulose Chally, Abdul Azeez, Shrikant Patel, Arifa Bakerywala, Heena Shaikh

**Affiliations:** 1 Department of Urology, Manipal Hospital Malleshwaram, Bengaluru, Karnataka, India; 2 Department of Urology, Consultant, Baby Memorial Hospital, Calicut Kerala, India; 3 Private practitioner, Shree ram dental clinic and implant centre, Bhavnagar, Gujarat, India; 4 Department of Pediatric dentistry, Tufts University School of Dental Medicine, United States of America; 5 Department of Healthcare Leadership, Trinity Western University, Canada

**Keywords:** Clear cell carcinoma, renal cell carcinoma, neutrophil-lymphocyte ratio, survival

## Abstract

The features of tumour in clear cell - renal cell carcinoma are evaluated using neutrophil - lymphocyte ratio for its prognosis. Hence, 186 clear cell-renal cell carcinoma patients with documented neutrophil lymphocyte ratio were obtained. Depending on the features of the lesion, patients underwent either a partial or radical nephrectomy and characteristics were studied in relation to normal or high neutrophil - lymphocyte ratio with a cut-off of 2.7. Of the 186 patients studied, 131 had a normal neutrophil lymphocyte ratio (<2.7), while 55 presented with an elevated neutrophil lymphocyte ratio (≥2.7). Elevated neutrophil lymphocyte ratio was significantly associated with both tumor size and renal vein invasion, with a p-value of less than 0.001. Thus, the neutrophil lymphocyte ratio is a valuable metric for assessing renal vein extension and predicting tumour size.

## Background:

Renal cell carcinoma is an immunogenic cancer characterised by extensive vascularization and substantial infiltration of various immune cells. Consequently, contemporary therapeutic techniques employ cancer immunotherapy, anti-antigenic agents, or a combination of both [1, 2, 3- 4]. With a death rate of up to 40%, this urinary system tumour is extremely malignant [5, 6]. The clear cell renal cell carcinoma is insensitive to traditional chemotherapy or radiation therapy [7]. Globally, there has been an increase in the prevalence and unintentional discovery of renal cell carcinoma in asymptomatic patients [8, 9]. Currently, medical imaging examinations are the primary method used to detect clear cell renal cell carcinoma because the majority of patients with this type of cancer present with unusual clinical signs. About 25% of patients have metastases at the time of diagnosis and nearly 50% of kidney tumours are found by accident [10, 11]. Inflammation and cancer are closely related and individuals with cancer experience will have both systemic and localised alterations in inflammatory markers that include modifications to the erythrocyte sedimentation rate, changes in the number of neutrophils, lymphocytes and neutrophil to lymphocyte ratio in peripheral blood cells, as well as changes to their phenotypes and gene expression patterns. Additionally, there are changes to the level of acute-phase proteins such as, C-reactive protein, fibrinogen, albumin and transferrin and serum inflammatory cytokines [12, 13-14]. Recent data indicates that patients with hepatocellular carcinoma and colorectal cancer are the ones in whom Neutrophil Lymphocyte Ratio acquired its prognostic significance [15, 16 and 17]. Individuals with renal cell carcinoma who have higher pre-treatment Neutrophil Lymphocyte Ratio levels might have worse clinical outcomes [18]. The neutrophil-lymphocyte ratio is the ratio of the neutrophil to lymphocyte count [19]. Increased neutrophil-to-lymphocyte ratios in cancer patients may indicate compromised cell-mediated immunity as well as neutrophilia and lymphopenia. As a result, Neutrophil Lymphocyte Ratio is regarded as a reliable predictive biomarker for some tumours, such as genitourinary or gut malignancies [19, 20, 21, 22- 23]. Therefore, it is of interest to assess the relevance of neutrophil lymphocyte ratio in tumor characteristics and survival in clear cell-renal cell carcinoma.

## Materials and Methods:

Present study was conducted at, Baby Memorial Hospital located in Calicut, Kerala, India. It was hospital-based retrospective study over a period of 15 years from January 2005 to March 2019. The research focused on 186 clear cells - renal cell carcinoma patients registered for treatment under the Department of Urology. Ethical clearance for the study was obtained from the (Institutional Ethical and Review Committee with reference number: BMH/Aca/DNB/Uro/EC/1756/08; Dated: 12th April 2019) and also collected permission from the patients and hospital administrations for retrieving and using the hospital data. Our study involved patients with clear cell renal cell carcinoma and their documented Neutrophil Lymphocyte Ratio was sourced from the hospital's information system. The selection criteria included a histologic diagnosis of metastatic or locally advanced unrespectable renal cell carcinoma, clear cell histology and an age of over 18. Patients requiring emergency surgery, those with compromised cardiac, lung, liver, or kidney function and individuals over the age of 85 were excluded from the study. Patients were subjected to Partial or Radical Nephrectomy as per the lesion characteristics. Patients were followed and collected clinical history, blood investigations and imaging studies. A cut-off point was used to stratify the study population into Neutrophil Lymphocyte Ratio low (<2.7) and Neutrophil Lymphocyte Ratio high (≥2.7) categories and comparisons were made. Data was entered in to Microsoft Excel and analysis was done using Statistical Package for Social Sciences statistics 25.0 version (IBM Corp., Armonk, New York, USA). Categorical variables were presented in proportions and continuous variables were presented in mean with standard deviation. A comparison of qualitative factors was done by using the chi-square test and continuous data was evaluated using the independent student t test. A significance level that was deemed statistically significant was established as a p value less than 0.05.

## Results:

Among the 186 cc-renal cell carcinoma patients, 161 (86.6%) were male and 25 (13.4%) were females, making a ratio of 6.4:1. The mean age of our patient cohort was 57.1 ± 8.64 years (Range: 32 - 74 years). Symptomatic presentation was seen in 89(47.8%) cases, while incidental detection was made in 97 (52.2%) cases. Right sided tumour was more common [n=103, 55.4%], than left side [n=82; 44.1%], with only one (0.5%) case having bilateral synchronous tumour. Average tumour size was 7.4 ± 2.27 cm (Range: 1 to 12 cm). Median hospital stay was 9 days, with an inter-quartile range of6 to 22 days). Nearly, 133 (71.5%) patients had radical nephrectomy and remaining 53 (28.5%) had partial nephrectomy (Figure 1 - see PDF). Around 131 (70.4%) patients had <2.7 Neutrophil-Lymphocyte ratios and remaining 55 (29.6%) had ≥2.7 (Figure 2 - see PDF). Comparisons of tumour characteristics with Neutrophil Lymphocyte Ratio cut off were presented in Table 1. The average tumour size in patients with Neutrophil Lymphocyte Ratio <2.7 was 8.1 cm (SD: 3.74), which was significantly larger compared to those with Neutrophil Lymphocyte Ratio ≥2.7, whose average tumour size was 6.0 cm (SD: 2.96). Positive for capsular invasion among patients with Neutrophil Lymphocyte Ratio <2.7 and Neutrophil Lymphocyte Ratio ≥2.7 was seen in 79 (60.3%) and 30 (54.5%) patients respectively. Positivity at Sinuses were seen in 47 (35.9%) patients with Neutrophil Lymphocyte Ratio <2.7 and 27 (49.1%) with Neutrophil Lymphocyte Ratio ≥2.7. Renal vein extension was seen in 15 (11.5%) patients with Neutrophil Lymphocyte Ratio <2.7and 20 (36.4%) patients with Neutrophil Lymphocyte Ratio ≥2.7, showing a significantly higher occurrence in the latter group. Inferior vena cava extension was seen in only 6 (4.6%) patients with Neutrophil Lymphocyte Ratio <2.7and 5 (9.1%) patients with Neutrophil Lymphocyte Ratio ≥2.7. Lymph node positivity was also seen in 8 (6.1%) patients with Neutrophil Lymphocyte Ratio <2.7 and 5 (9.1%) patients with Neutrophil Lymphocyte Ratio ≥2.7. Metastasis was seen in16 (12.2%) patients with Neutrophil Lymphocyte Ratio <2.7 and 11 (20%) patients with Neutrophil Lymphocyte Ratio ≥2.7. Most of the cases either with Neutrophil Lymphocyte Ratio <2.7 or Neutrophil Lymphocyte Ratio ≥2.7 had grade 2 tumour under Fuhrman grading. Around 5 (3.8%) patients with Neutrophil Lymphocyte Ratio <2.7 and 6 (10.9%) patients with Neutrophil Lymphocyte Ratio ≥2.7 were died during follow-up. Though death rate was higher in patients with Neutrophil Lymphocyte Ratio ≥2.7, but the difference was not statistically significant.

## Discussion:

Renal cell carcinoma accounts for 2.4% of all cancer diagnoses and has become more common during the past 20 years worldwide [24, 25]. In a small percentage of individuals, surgery can be curative when the disease is still in its early stages. Systemic therapy is necessary for advanced and metastatic stages, nevertheless. Renal cell carcinoma is a malignancy that is resistant to treatment and highly immunogenic [26, 27-28]. The ratio of neutrophils to lymphocytes (NLR) illustrates how innate and adaptive immunological activities are dynamically balanced. Consequently, a high Neutrophil Lymphocyte Ratio indicates immunological discomfort and persistent inflammation [29, 30]. A Neutrophil Lymphocyte Ratio of ≥3 indicates elevated readings, which are considered unhealthy [31, 32].The Neutrophil Lymphocyte Ratio is a widely used biomarker for inflammatory states, inflammatory disorders and a variety of malignant tumours. It is easy to use, affordable and accessible [33, 34]. Both an increase in circulating neutrophils and a decrease in systemic inflammatory lymphocytes lead to an increase in Neutrophil Lymphocyte Ratio. In the meanwhile, Neutrophil Lymphocyte Ratio has demonstrated effectiveness as a substitute marker for systemic inflammation in cancers, end-stage renal disease, diabetes and critically unwell individuals [35, 36]. Arda *et al.* showed that immunosuppression and inflammation play a role in the aetiology of cancer and that an elevated Neutrophil Lymphocyte Ratio is linked to unfavourable cancer outcomes [37]. Neutrophil Lymphocyte Ratio has been found by Pichler *et al.* to be a useful signal for renal cell cancer preoperative diagnosis [38]. According to research by Ohno *et al.* there is a substantial correlation between Neutrophil Lymphocyte Ratio and a higher death rate from colorectal cancer. Neutrophil Lymphocyte Ratio is a low-cost, user-friendly and reliable clinical technique for colorectal cancer prognostic prediction [39]. Neutrophil Lymphocyte Ratio is a significant predictive factor for overall survival (OS) and disease-free survival (DFS) following R0 resection for gastric cancer, as demonstrated by Ramsey *et al.* [40] however its critical value is yet unknown. Pichler *et al.* [38] discovered that preoperative Neutrophil Lymphocyte Ratio increase was linked to a poor overall survival but not to cancer-specific outcomes in a sizable, validated European investigation of Neutrophil Lymphocyte Ratio pre-treatment prognosis in 678 patients with renal cell carcinoma.

Survival is better for patients with lower neutrophil lymphocyte ratio, but didn't have significance. However, Vincenzo *et al.* study had got significant association of elevated neutrophil lymphocyte ratio with higher Overall Survival and Progression-Free Survival (Overall Survival pooled Heart Rate 1.80; 95%CI: 1.61-2.00; I2 45%; Progression-Free Survival pooled Heart Rate of 1.69; 95%CI: 1.42-2.01; I2 81%). Furthermore, Neutrophil Lymphocyte Ratio is a readily available biomarker for renal cell carcinoma that can be utilised to assess prognosis. The Neutrophil Lymphocyte Ratio may be a helpful diagnostic biomarker for renal cell cancer in the preoperative staging, as specified in Selahattin *et al.* study [24]. The significance of higher Neutrophil Lymphocyte Ratio (≥2.7) in patients with clear cell RCC is correlated well with overall survival.

## Conclusion:

The neutrophil lymphocyte ratio is a valuable metric for assessing the characteristics and prognosis of various solid tumours, including clear cell - renal cell carcinoma. We show the importance of elevated neutrophil lymphocyte ratio with involvement of renal vein extension even though having smaller tumour size, signifies elevated neutrophil lymphocyte ratio will be more migratory tumour than having large localised tumours. The survival also affected with elevated neutrophil lymphocyte ratio, though it wasn't statistically significant, but correlated will clinically.

## Source of funding:

There was no financial support concerning this work.

## Figures and Tables

**Table 1:** Comparison of tumour characteristics in relation with Neutrophil Lymphocyte Ratio cut-off

**Tumour characteristic**		**Neutrophil Lymphocyte Ratio**		**p-value**
		**<2.7 (n=131)**	**≥ 2.7 (n=55)**	
Tumour size in centimeters (Mean ± SD)		8.1 ±3.74	6.0 ± 2.96	<0.001; S
Capsular invasion	Positive	79 (60.3%)	30 (54.5%)	0.467; NS
	Negative	52 (39.7%)	25 (45.5%)	
Sinus	Positive	47 (35.9%)	27 (49.1%)	0.093; NS
	Negative	84 (64.1%)	28 (50.9%)	
Renal vein extension	Positive	15 (11.5%)	20 (36.4%)	<0.001; S
	Negative	116 (88.5%)	35 (63.6%)	
Inferior vena cava Extension	Positive	6 (4.6%)	5 (9.1%)	0.234; NS
	Negative	125 (95.4%)	50 (90.9%)	
Lymph node positivity	Positive	8 (6.1%)	5 (9.1%)	0.466; NS
	Negative	123 (93.9%)	50 (90.9%)	
Metastasis	Positive	16 (12.2%)	11 (20%)	0.169; NS
	Negative	116 (87.8%)	44 (80%)	
Fuhrman	Grade 1	12 (9.2%)	5 (9.1%)	0.088; NS
grade	Grade 2	67 (51.1%)	25 (45.5%)	
	Grade 3	48 (36.6%)	18 (32.7%)	
	Grade 4	4 (3.1%)	7 (12.7%)	
Mortality	Dead	5 (3.8%)	6 (10.9%)	0.061; NS
	Alive	126 (96.2%)	49 (89.1%)	
SD = Standard deviation;
S = Significant;
NS = Not Significant

## References

[1] Siegel R.L (2018). CA Cancer J Clin..

[2] Padala S.A (2020). World J Oncol..

[3] Heidegger I (2019). Front Oncol..

[4] Bamias A (2017). Oncologist..

[5] Song X.D (2020). J Int Med Res..

[6] Motzer R.J (2015). Lancet Oncol..

[7] Hötker A.M (2016). AJR Am J Roentgenol..

[8] Weikert S, Ljungberg B (2010). World J Urol..

[9] Choi J.B (2011). Korean J Urol..

[10] Kwon R.J (2017). PLoS One..

[11] Cheng S.K, Chuah K.L (2016). Arch Pathol Lab Med..

[12] Casamassima A (2005). J Urol..

[13] Komai Y (2007). BJU Int..

[14] McMillan D.C (2003). Br J Surg..

[15] Ohno Y (2010). J Urol..

[16] Li M.X (2014). Int J Cancer..

[17] Xiao W.K (2014). BMC Cancer..

[18] Ohno Y (2014). Int J Clin Oncol..

[19] Chen L (2021). Medicine (Baltimore)..

[20] Yang Y (2018). Biosci Rep..

[21] Faria S.S (2016). Ecancermedicalscience..

[22] Patel A (2020). Clin Cancer Res..

[23] Cordeiro M.D (2022). Clin Genitourin Cancer..

[24] Caliskan S (2018). Folia Med (Plovdiv)..

[25] Ziegelmüller B.K (2018). Urologe A..

[26] Isaac V (2016). Medicine (Baltimore)..

[27] Otunctemur A (2016). Int Braz J Urol..

[28] Allenet C (2022). Cancers (Basel)..

[29] Afari M.E, Bhat T (2016). Expert Rev Cardiovasc Ther..

[30] Pirozzolo G (2019). J Thorac Dis..

[31] Guthrie G.J (2013). Crit Rev Oncol Hematol..

[32] Turkmen K (2012). Ren Fail..

[33] Eochagáin Ni-A (2018). Anaesthesia..

[34] Nunno V.D (2019). Immunotherapy..

[35] Palin R.P (2018). Ann R Coll Surg Engl..

[36] Mellor K.L (2018). J Gastrointest Cancer..

[37] Arda E (2018). Cureus..

[38] Pichler M (2013). Br J Cancer..

[39] Ohno Y (2012). J Urol..

[40] Ramsey S (2008). BJU Int..

